# A Law Regulating the Practice of Medicine and Surgery

**Published:** 1874-06

**Authors:** 


A LAW REGULATING THE PRACTICE OF MEDICINE AND SURGERY IN THE STATE OF NEW YORK, recently passed bp the Legislature, and which has received executive sanction:

CHAPTER 436.

AN ACT to Regulate the Practice of Medicine and Surgery in the State of New York.

PASSED May 11, 1874.

*The People of the State of New York, represented in Senate and Assembly, do enact as follows:*

SECTION 1. Every practitioner of medicine or surgery in this State, excepting licentiates or graduates of some medical society or chartered school, shall be required and they are hereby commanded to obtain a certificate from the censors of some one of the several medical societies of this State either from the county, district or State society; which certificate shall set forth that said censors have found the person to whom it was issued qualified to practice all the branches of the medical art mentioned in it. And such certificate must be recorded in a book provided and kept for the purpose by the county clerk of each county in the State.

SEC. 2. The censors of each medical society aforesaid shall notify all practitioners of medicine and surgery of the terms and requirements of this act, and shall request such person, so notified, to comply with those requirements within thirty days after such notification; and if such persons shall not, within the time specified in the notice, or within such further time as may be allowed by special arrangemtnt with said censors, not exceeding ninety days, comply with the requirements herein made of physicians or surgeons, as the case may be, such persons shall thereafter be subject to all the provisions and penalties prescribed by this act for any violation of the same, and the president of the society making such request, shall and he is hereby required to at once commence the proceedings authorized by this act against such person.

SEC. 3. It is hereby declared a misdemeanor for any person to practice medicine or surgery in this State, unless authorized so to do by a license or diploma from some chartered school, State Board of Medical Examiners, or medical society, or who shall practice under cover of a medical diploma illegally obtained; and any person found guilty of such a misdemeanor shall, for the first offense, be fined not less than fifty nor more than two hundred dollars; for any subsequent offense, not less than one hundred nor more than five hundred dollars, or by imprisonment not less than thirty days, or by both imprisonment and fine; and all such fines shall go into the county treasury of the county bringing such action.

At the last meeting of the Erie County Medical Society (June 9), the board of censors was directed to prepare a list of all persons in Erie county practicing in violation of this law, and on the completion of such list to notify the President, who is to call a special meeting of the Society to take action in the matter.

